# Generalized morphea following the COVID vaccine: A series of two patients and a bibliographic review

**DOI:** 10.1111/dth.15709

**Published:** 2022-07-20

**Authors:** Javier Antoñanzas, Nuria Rodríguez‐Garijo, Ángela Estenaga, Ana Morelló‐Vicente, Agustín España, Leyre Aguado

**Affiliations:** ^1^ Department of Dermatology University Clinic of Navarra, School of Medicine, University of Navarra Pamplona Spain

**Keywords:** COVID, cross‐reactivity, immunorelated, morphea, vaccination

## Abstract

The appearance of morphea after vaccination has been reported to date as single and deep lesions that appear exactly at the site of the skin puncture. It was therefore postulated that the origin could be the trauma related to the injection. The aim of this article is to review the various hypotheses offered in the published literature about generalized morphea following vaccination. We present two cases of generalized morphea after COVID‐19 vaccination and review the published literature on immune‐related cutaneous reactions. As previously reported, antigenic cross‐reactivity between vaccine spike proteins and human tissues could cause certain immune‐mediated diseases, including generalized morphea. Herein we report two cases of generalized morphea probably induced by the COVID‐19 vaccine, given the temporal relationship with its administration. In summary, environmental factors such as vaccination against SARS‐COV‐2 could induce an immune system dysregulation, which would have an important role in the pathogenesis of morphea. We present two cases of generalized morphea probably induced by the COVID‐19 vaccine, given the time elapsed between vaccination and the onset of the skin lesions.

## 1 INTRODUCTION

Some dermatoses may appear secondary to vaccination. For example, cases of close temporal relationships between the development of granuloma annulare and the antitetanic vaccine have been reported,^1^ and also patients with lichen planus,^2^ bullous pemphigoid^3^ and mast cell tumors^4^ after the hepatitis B vaccination. In all these cases, the skin lesions appeared a few weeks after receiving the vaccines and developed exactly at the site where the vaccines were injected, suggesting a possible causal relationship.^5^ The appearance of morphea after vaccination is rare and the cases reported to date appear in predisposed patients, most of whom were children. Clinically, morphea lesions usually present with brownish, indurated or atrophic plaques at the injection site, which can become generalized and even cause functional impairment. The exact mechanism by which this occurs is unknown, but it could be due to antigenic cross‐reactivity.^6^ Herein, we present two patients with generalized morphea lesions after receiving the COVID‐19 vaccine.

## 2 CASE 1

**FIGURE 1:**
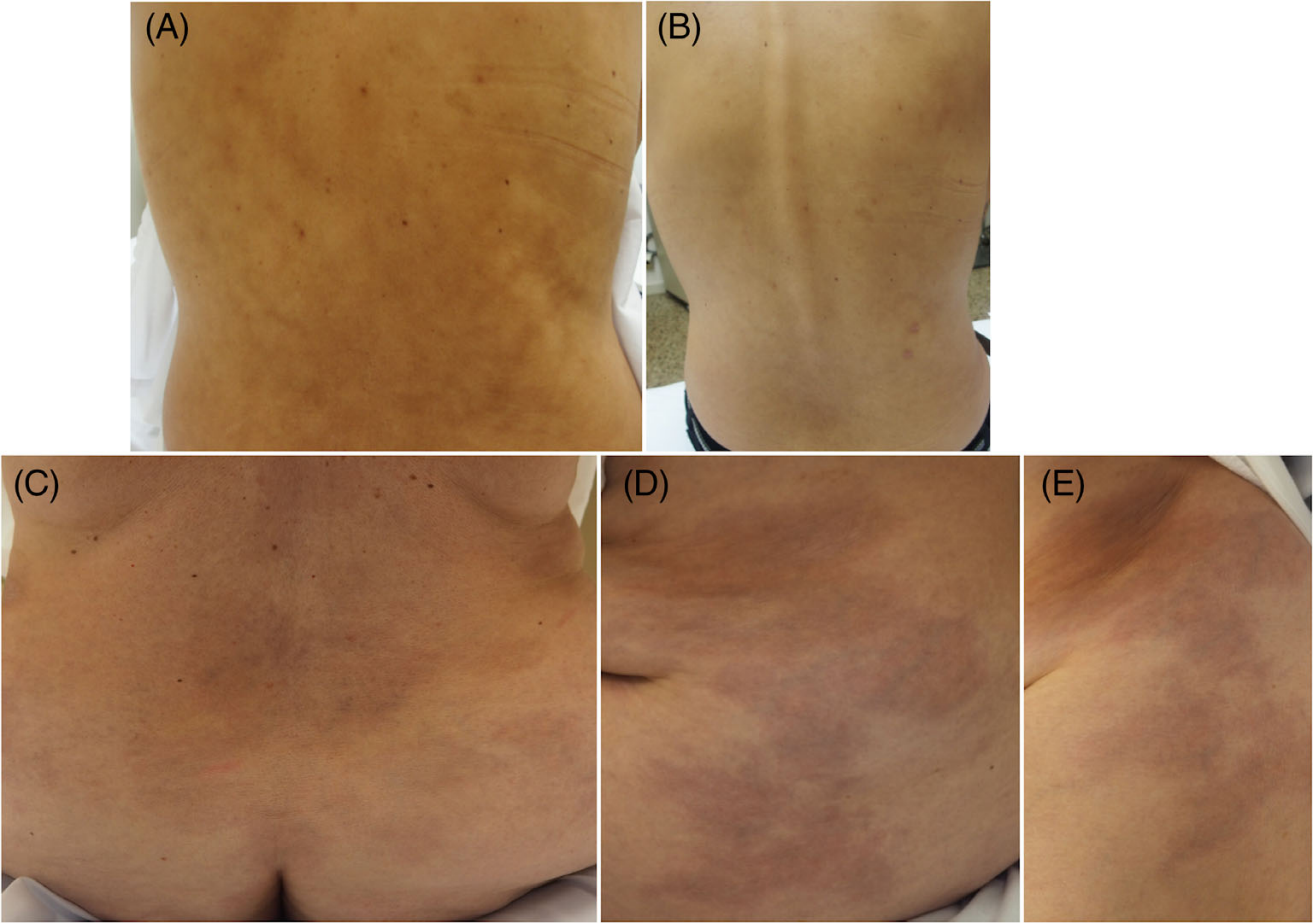
Generalized morphea lesions. (A) Brownish patches of indurated consistency localized on the back of the patient. (B) Resolution of morphea lesions after treatment with betamethasone and topical calcipotriol. (C) Brownish plaques of indurated consistency localized on the thighs and the back of the patient (D)

A 45‐year‐old woman with no relevant medical history, presented with patchy oval indurated lesions on her back. The patient was taking no new medications and had no systemic symptoms. A blood test for autoantibodies was negative. Two weeks earlier she received the first dose of the mRNA‐1273 COVID‐19 vaccine. A skin biopsy was performed which revealed a normal epidermis with thick collagen bundles. Given the suspicion of generalized morphea induced by the COVID‐19 vaccine, treatment with betamethasone and topical calcipotriol was begun and the patient showed great improvement (Figure 1) with no recurrences after 6 months. The patient experienced no problems when she received the second dose in the vaccination schedule and no longer presented cutaneous side effects.

## 3 CASE 2

**FIGURE 2:**
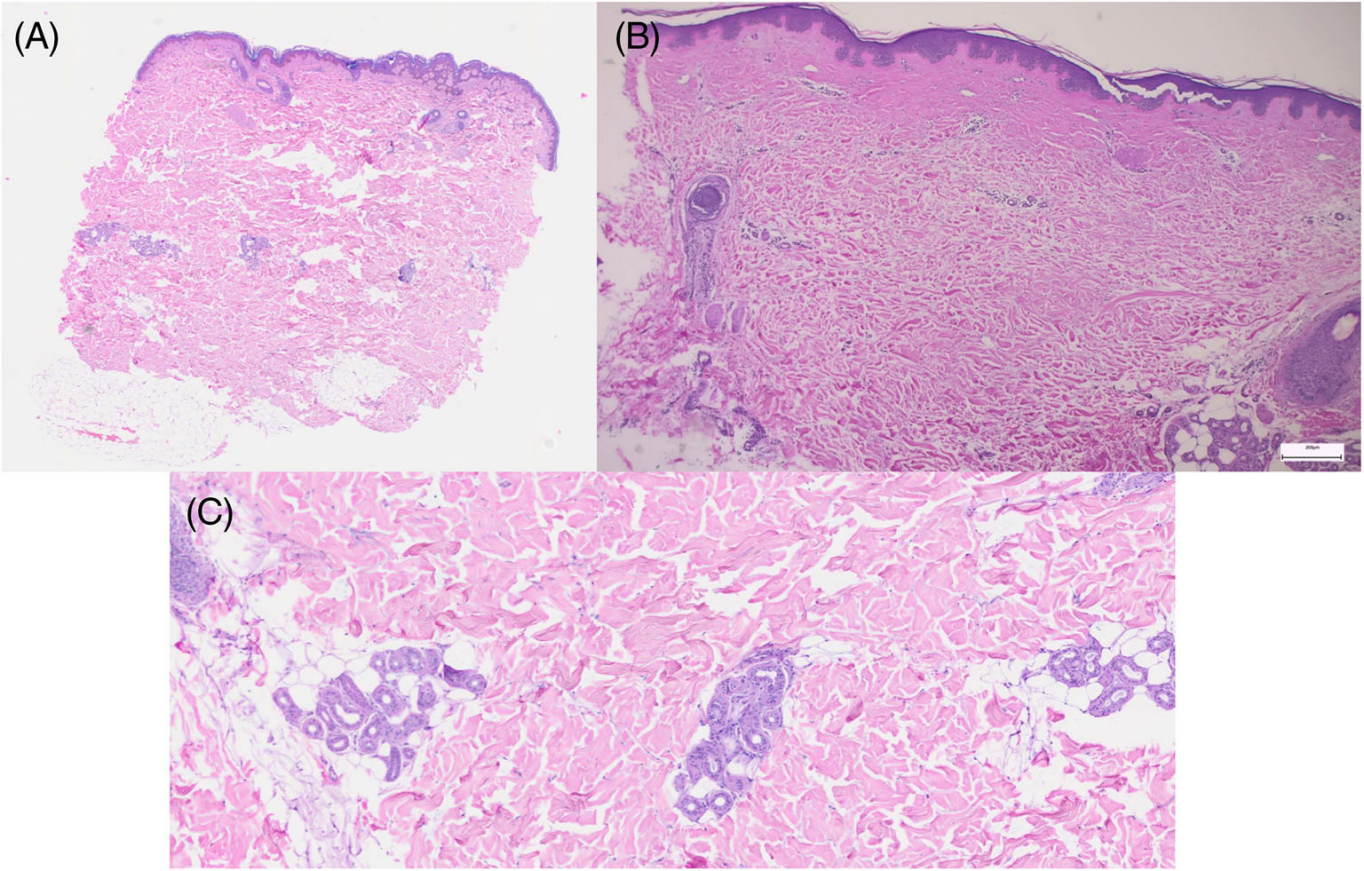
Histological findings. (A–C) Normal epidermis with mild perivascular inflammatory infiltrate and thick dermal collagen bundles

A 52‐year‐old woman with no relevant medical history presented with an approximately one‐month history of indurated brownish patches on the abdomen and thighs (Figure 1). About 6 weeks before of the onset of the rash, she received the second dose of the BNT162b2 COVID‐19 vaccine. Histological study showed an unaltered epidermis with thickening of the collagen bundles of the reticular dermis (Figure 2). A blood test also ruled out ANA and ANCA autoantibodies. With the suspicion of generalized morphea induced by the COVID‐19 vaccine, treatment with topical corticosteroids was begun. Due to the lack of improvement, oral methotrexate 15 mg/m^2^/week was initiated and lesions were seen to regress.

Morphea lesions secondary to vaccines are usually deep and solitary plaques and appear in the area where the skin was punctured; however, over time they can become generalized and affect other locations, leading to functional impairment in some joints.^7^ The etiopathology of the reaction is unknown, but trauma has been implicated as an important trigger. Additionally, exposure to chemicals, including organic solvents, toxic oil, l‐tryptophan, silica and silicone, or the injection of chemicals or medications, such as paraffin, bleomycin, methysergide, pentazocine, vitamin K1, and corticosteroids, have also been associated as causes of morphea and sclerodermiform lesions.^2^

The appearance of morphea after vaccination is rare in relation to the large percentage of the population that receives vaccines throughout life. The fact that it has been described with different types of vaccines suggests that a genetic predisposition is involved in the pathogenesis of the disease.^5^ The administration of the vaccines could induce an immune response against the specific antigen administered but also against other nonspecific antigens, leading to the release of some cytokines and the activation of the inflammatory cascade.

Given the current pandemic situation due to the SARS‐COV2 virus, mRNA‐based vaccines consisting of a sequence‐optimized mRNA that encodes the peak glycoprotein encapsulated in lipid nanoparticles have been developed. These vaccines, have shown both the production of antibodies against the virus glycoprotein and also the development of cellular responses, mainly biased towards CD4 + Th1 cells.^8^

Some cutaneous adverse effects have been observed after COVID‐19 vaccination and include type I hypersensitivity reactions such as urticaria, angioedema and anaphylaxis, type IV hypersensitivity reactions such as COVID arm, filler reactions and inflammatory lesions at sites of previous radiation. Other autoimmune‐mediated skin findings after COVID‐19 vaccination are lichenoid dermatitis, leukocytoclastic vasculitis, lupus erythematosus, immune thrombocytopenia and finally and more commonly, morbilliform rash and erythema multiforme.^9^, ^10^

The molecular similarity between SARS‐CoV‐2 vaccines (spike protein sequences) and some human tissue proteins such as transglutaminase, mitochondria, actin, and myelin basic protein could explain the appearance of skin reactions after vaccination.^6^ These reactions, mediated by T cells, have been also attributed to vaccine ingredients such as neomycin or thimerosal and to delayed‐type hypersensitivity reactions against the polyethyleneglycol excipient.^11^ An additional mechanism could be the injection‐related trauma that causes damage to the vascular endothelia leading to the tissue hypoxia, which is characteristic of morphea lessions.^2^

To date, some cases of morphea induced by COVID‐19 infection^12^, ^13^ have reported although there are only isolated cases reporting morphea triggered by the COVID‐19 mRNA vaccines.^6^, ^14^ In one of these patients, morphea lesions appeared 4 weeks after the second dose of the BioNTech COVID‐19 vaccine and the immunohistochemical study performed was positive for the anti‐SARS‐CoV‐2 spike glycoprotein 1 monoclonal antibody in the vessels, the inflammatory cells and the secretory portions of the eccrine sweat glands. However, the authors concluded that the positive immunostaining may have been the result of cross‐reactivity, and the stained proteins may have been tissue components.^6^

In the first patient we report, the generalized morphea lesions appeared two weeks after receiving the first dose of the vaccine, while in the second case the lesions appeared six weeks after the second dose. Devon et al.^5^ reported adverse skin effects after vaccination in a series of 180 patients, with 38/180 (21%) presenting reactions after the first dose only, 113/180 (63%) after the second dose only, and 29/180 (16%) to both doses. Therefore, of the 67 patients who had cutaneous reactions to the first dose, only 29 (43%) also had reactions to the second dose. In the first case we present, the generalized morphea lesions were treated successfully and no new lesions appeared after the second dose, whereas in the second case, the morphea lesions only appeared after the second dose.

The absence of a relevant medical history, laboratory abnormalities and the close temporal relationship with the administration of the COVID‐19 vaccine suggest a possible causal relationship between the morphea and the COVID‐19 vaccination. To the best of our knowledge, the Diphtheria‐Tetanus‐Pertussis vaccine, triple viral, hepatitis B and influenza vaccines have also been suggested as possible causes of morphea as have recently COVID‐19 vaccines.^14^

In conclusion, we present two cases of generalized morphea probably induced by the COVID‐19 vaccine, given the temporal relationship with vaccine administration. Further research is needed to better understand the pathophysiological basis of the disease and to better assess the true prevalence of the COVID‐19 vaccination skin reactions.

## AUTHOR CONTRIBUTIONS

**Javier Antoñanzas**: conception and design, drafting of the manuscript and critical review of the intellectual content. **Nuria Rodríguez‐Garijo**: conception and design, critical review of the content and final approval of the version to be published. **Ángela Estenaga**: critical review of the content. **Ana Morelló Vicente**: critical review of the content. **Agustín España**: conception and design, critical review of the content and final approval of the version to be published. **Leyre Aguado**: conception and design, critical review of the content and final approval of the version to be published. All the authors that appear in this article have contributed directly to its realization.

## FUNDING INFORMATION

None.

## CONFLICT OF INTEREST

The authors declare that they have no source of funding or conflict of interest.

## Data Availability

Data sharing not applicable to this article as no datasets were generated or analysed during the current study.
